# Understanding childhood obesity in Pakistan: exploring the knowledge, attitudes, practices of mothers, and influential factors. A cross-sectional study

**DOI:** 10.3389/fpubh.2024.1475455

**Published:** 2024-11-06

**Authors:** Muhammad Hudaib, Laraib Hussain, Laiba Nazim, Sumaira Mohi Uddin, Muhammad Usama Jamil, Shireen Qassim Bham, Hurais Malik, Abdul Rehman, Usaid Malik, Manahil, Ahmad Umais Ahad, Sanila Mughal, Mohammed Mahmmoud Fadelallah Eljack

**Affiliations:** ^1^Fazaia Ruth Pfau Medical College, Karachi, Pakistan; ^2^Baqai Medical University, Karachi, Pakistan; ^3^Nishtar Medical University, Multan, Pakistan; ^4^Department of Pediatrics, Fazaia Ruth Pfau Medical College, Karachi, Pakistan; ^5^Department of Pediatrics, CMH Multan Institute of Medical Sciences, Multan, Pakistan; ^6^Department of Obstetrics and Gynaecology, Pakistan Institute of Medical Sciences, Islamabad, Pakistan; ^7^Department of Medicine, Pakistan Institute of Medical Sciences, Islamabad, Pakistan; ^8^Dow Medical College, Karachi, Pakistan

**Keywords:** childhood obesity, knowledge, attitudes, practices, mothers, public health, health education, Pakistan

## Abstract

**Background:**

Childhood obesity is a significant public health issue with far-reaching implications. The World Health Organization reported that in 2020, around 38 million children under five were overweight or obese globally, and in 2016, 340 million children and adolescents aged 5–19 were affected. In Pakistan, the situation is alarming; 66.9% of school-going children in Punjab were overweight, and 5.8% were obese in 2022. This study explores mothers’ knowledge, attitudes, and practices regarding childhood obesity in Pakistan and identifies factors contributing to this epidemic. Maternal perspectives are crucial as they significantly influence children’s dietary habits, physical activity, and attitudes toward food and body image.

**Methods:**

A cross-sectional study was conducted from November 2023 to January 2024 at four medical centers: Fazaia Ruth Pfau Medical College Hospitals Karachi, Baqai Medical University Karachi, and Nishtar Medical University Multan. The study included 191 mothers with children aged 5 to 15 years. Data were collected using a structured questionnaire on sociodemographic characteristics, knowledge, attitudes, practices, and perceptions of childhood obesity. IBM-SPSS version 26.0 was used for data analysis, employing statistical tests like Kruskal-Wallis, Mann–Whitney U, Spearman, or Kendall Tau correlation to examine associations.

**Results:**

Among the 191 mothers, 48.7% had education beyond intermediate, and 86.9% were housewives. The children’s BMI distribution showed that 27.7% were obese and 21.5% overweight. Mothers had moderate knowledge (60.5%) about childhood obesity; 75.4% recognized its long-term health risks, and 62.8% associated it with diabetes. Attitudes were generally positive, with a 78.5% average score. Most mothers (73.8%) believed obesity could be controlled and had healthy practices (70.1% average score). However, 96.9% reported witnessing stigmatization of obese children, and 79.6% felt pressured by relatives about their child’s weight.

**Conclusion:**

The findings indicate that while Pakistani mothers have moderate knowledge and positive attitudes toward childhood obesity, their practices are influenced by educational and socioeconomic factors. Addressing these disparities, enhancing public health initiatives, and mitigating stigmatization could improve childhood obesity management in Pakistan.

## 1 Introduction

Childhood obesity is a pressing public health concern with far-reaching implications for the well-being of children and societies at large (1). Defined as an excessive accumulation of body fat, childhood obesity has witnessed a global surge in prevalence over recent decades (2). The global prevalence of childhood obesity has risen dramatically in recent years, emerging as one of the most significant public health challenges of our time. According to the World Health Organization (WHO), the worldwide prevalence of childhood obesity has reached alarming proportions, with approximately 38 million children under the age of five classified as overweight or obese in 2020, and a staggering 340 million children and adolescents aged 5–19 facing this issue in 2016 (3). Pakistan, in particular, has not been immune to this growing epidemic. Recent research conducted in 2022 on school-going children in Punjab revealed that a staggering 66.9% of children were overweight, with an additional 5.8% classified as obese (4).

These disconcerting statistics carry substantial implications for the long-term health and prosperity of affected individuals. Childhood obesity often persists into adulthood and is linked to an increased risk of chronic diseases, such as diabetes, cardiovascular diseases, and specific cancers (5, 6). Furthermore, it is essential to acknowledge the socio-economic ramifications of obesity, particularly for developing nations like Pakistan (7). In response to this alarming trend, research endeavors have intensified to comprehend the multifaceted determinants of childhood obesity and to formulate effective prevention and intervention strategies. Among the myriad factors influencing childhood obesity, the role of mothers, as primary caregivers and nurturers within families, has garnered significant attention. Mothers wield a pivotal influence in shaping their children’s dietary choices, physical activity habits, and attitudes toward food and body image (8). Their perceptions, beliefs, and practices concerning childhood obesity represent critical determinants of the environment in which their children grow and develop (9). These maternal perspectives can either facilitate the adoption of a healthy lifestyle or inadvertently contribute to the risk of obesity development (10).

However, within the Pakistani context, research exploring factors contributing to obesity is limited, and investigations into the role of mothers in this context are notably scarce. This study seeks to address this gap by conducting a comprehensive examination of mothers’ perceptions of childhood obesity. It aims to shed light on their attitudes, knowledge, and practices concerning this critical issue. By delving into the intricacies of maternal perspectives, this research endeavors to provide a nuanced understanding of the underlying factors driving childhood obesity in Pakistan.

## 2 Methodology

### 2.1 Ethical considerations

The study obtained ethical approval for the study was obtained from the four respective Institutional Review and Ethics Boards, Fazaia Ruth Pfau Medical College Hospitals Karachi, Pakistan (10–11-23), Baqai Medical University, Karachi, Pakistan (02–08-23) and Nishtar Medical University, Multan, Pakistan (28–08-23) (FRPMC Ref No: IRB/45, BMU Ref No: BMU-IREB/03–2023, NMU Ref No:13131/NMU). The research adhered to ethical standards outlined in the Declaration of Helsinki of the World Medical Association for trials involving humans, as per the authors’ statements. The consent form was given to the participants which contained information about the study’s purpose, the voluntary nature of their participation, and the measures taken to protect their privacy and confidentiality. Participants were made aware of their right to withdraw from the study at any point without consequences. Additionally, it contained a debriefing statement, promoting transparency and communication. Data security measures were rigorously implemented to safeguard participant information. To ensure the privacy and confidentiality of participants, all data collected was de-identified and stored securely. Personal information such as names, contact details, or other identifiers was not linked to the collected data. Only the research team had access to the data, and any published results or reports would not contain any information that could identify individual participants. The data was stored on secure servers and password-protected computers.

### 2.2 Study design

A cross-sectional study was conducted between November 2023 and January 2024 at four centers Fazaia Ruth Pfau Medical College (FRPMC) Hospitals Karachi, Baqai Medical University (BMU) Karachi, and Nishtar Medical University (NMU) Multan to assess the knowledge, attitudes, and practices of Pakistani mothers regarding childhood obesity and the factors affecting it. The study focused on mothers with children aged between 5 to 15 years who were seeking healthcare services at the selected hospitals. Mothers with children aged less than 5 years or older than 15 years were excluded from participation. The children included were healthy with no history of chronic infection and immunization was up-to-date as per the Expanded Program of Immunization (EPI) schedule of the country. Respondents had the option to provide anonymous or non-anonymous responses based on their comfort and preference. The minimum sample size *n* = 76 was calculated by the WHO sample size calculator 2.0, using one sample situation: 1.1 Estimate a population proportion with specified absolute precision, with 95% confidence interval, 0.05 margin of error, and 5.2% prevalence of childhood obesity in the province of Sindh (11). A total of 191 mothers and children were included in the study based on eligibility criteria. No imputation methods were employed, and all responses were retained for analysis. Informed consent was obtained from all participants. Interviewers were trained to conduct interviews using a standardized questionnaire, which was available in both English and Urdu languages to accommodate participants’ language preferences and ensure clear communication.

### 2.3 Questionnaire development and validation

Initially, a pool of potential questionnaire items was generated based on a thorough review of existing literature related to childhood obesity and maternal perceptions. These items were designed to measure knowledge, attitudes, and practices of Pakistani mothers regarding childhood obesity. To enhance content validity, three experts in the field were consulted to review the questionnaire. Each expert on the panel independently reviewed and rated each questionnaire item on a scale of 1 to 10, with 1 indicating “not relevant” and 10 indicating “highly relevant” to the construct under investigation. The experts were specifically instructed to consider the importance or relevance of each item to the research topic. The Content Validity Ratio (CVR) was calculated for each item by determining the proportion of experts who rated the item as essential or highly relevant (typically with a rating of 7 or above on the 1 to 10 scale). The formula used for CVR calculation is as follows:

CVR = (Number of experts rating item as essential or highly relevant) / (Total number of experts) (12).

A predetermined consensus threshold was established, where items achieving a CVR value equal to or greater than a specified threshold (0.7) were considered to have content validity and were retained for the final questionnaire. Items that fell below the consensus threshold underwent further review. If necessary, these items were revised based on expert feedback to enhance their relevance. Items that could not achieve consensus even after revision were removed from the questionnaire. After the content validation process, a pilot test was conducted by administering the questionnaire to a small sample of 20 individuals representing the target population to ensure face validity. This pilot test aimed to identify potential issues, such as confusing or ambiguous questions, response options, or formatting problems. Feedback from pilot test participants was collected and used to refine and improve the questionnaire. The reliability of the questionnaire was checked through internal consistency (Cronbach alpha = 0.66).

### 2.4 Measures

The questionnaire employed in this study was meticulously designed to comprehensively investigate various dimensions associated with childhood obesity. It was organized into distinct sections to facilitate a comprehensive exploration of the topic. The initial section included 10 items aimed at collecting sociodemographic and socioeconomic data from the participants. These items encompassed the following variables: mother’s age, background, level of education, employment status, monthly income, number of children under the mother’s care, number of individuals residing in the household, age of the target child, and child’s gender. Additionally, the weight and height of all participants aged 5–15 years were measured using calibrated digital scales and stadiometers with millimeter scales, respectively. Body Mass Index (BMI) was then calculated for each participant using the formula BMI = weight (kg) / height (m)^2^. BMI categorization depending upon the age and sex of the child was determined using WHO criteria for children and adolescents (13).

The second section of the questionnaire was designed to assess the knowledge of mothers regarding childhood obesity. This section included nine items, each addressing a specific aspect related to childhood obesity and its long-term implications. Respondents were presented with three response options (Yes, No, or Maybe) for the first eight questions, however, the final item of the knowledge section involved the presentation of seven images representing a spectrum of BMI categories, ranging from underweight to obese, each depicting infants of comparable age and gender-neutral appearance. Participants were instructed to select the image they perceived as representing the healthiest baby. This approach provided a nuanced understanding of maternal perceptions regarding a child’s weight and health. The total score for this section was 12. The third section comprised 10 items intended to gauge the attitudes and beliefs of respondents regarding childhood obesity. These included statements such as “In your opinion, can obesity be controlled?” and “Do you believe fruits are healthy?” Participants were asked to rate their agreement with each statement on a 5-point Likert scale with a total score of 50 for this section.

The fourth section of the questionnaire addressed practices related to childhood obesity. It featured nine items aimed at assessing individuals’ behaviors concerning childhood obesity. These items included statements such as “Does your child regularly consume butter?” and “How much time does your child spend using electronic devices?” Respondents were presented with various response options. The maximum score for this section was 29.

The fifth section consisted of four items designed to assess individuals’ perceptions regarding the stigmatization of obese children. These items included statements like “Do you believe that underweight children are stigmatized in Pakistan?” and “Have you ever witnessed or experienced the stigmatization of obese children?” Respondents were provided with two response options: Yes and No. In the final section, participants were queried about their sources of information related to childhood obesity.

### 2.5 Data analysis

Data were stored and analyzed using IBM-SPSS version 26.0; Counts with percentages were reported for outcomes on knowledge, attitude, and practice for childhood obesity, However, Mean with Standard Deviation (SD) was reported for computed scores on knowledge, attitude, and practice. Comparisons of these scores with general and demographic factors were made using the non-parametric Kruskal Wallis test and Mann–Whitney U test. Correlation analysis of the three variables with demographic variables was done using Spearman Correlation analysis or Kendall Tau Correlation analysis. Bar diagrams were used to display the distribution of the BMIs of the index children, for knowledge scores, and for the distribution of responses on the attitude section of the questionnaire. For all statistical analyses, a significance level of *p* < 0.05 was adopted as the threshold for determining statistical significance. Any *p*-values below this threshold were considered statistically significant. Pie charts were also used to give a graphical summary of the source of the information regarding childhood obesity.

## 3 Results

**Figure 1:**
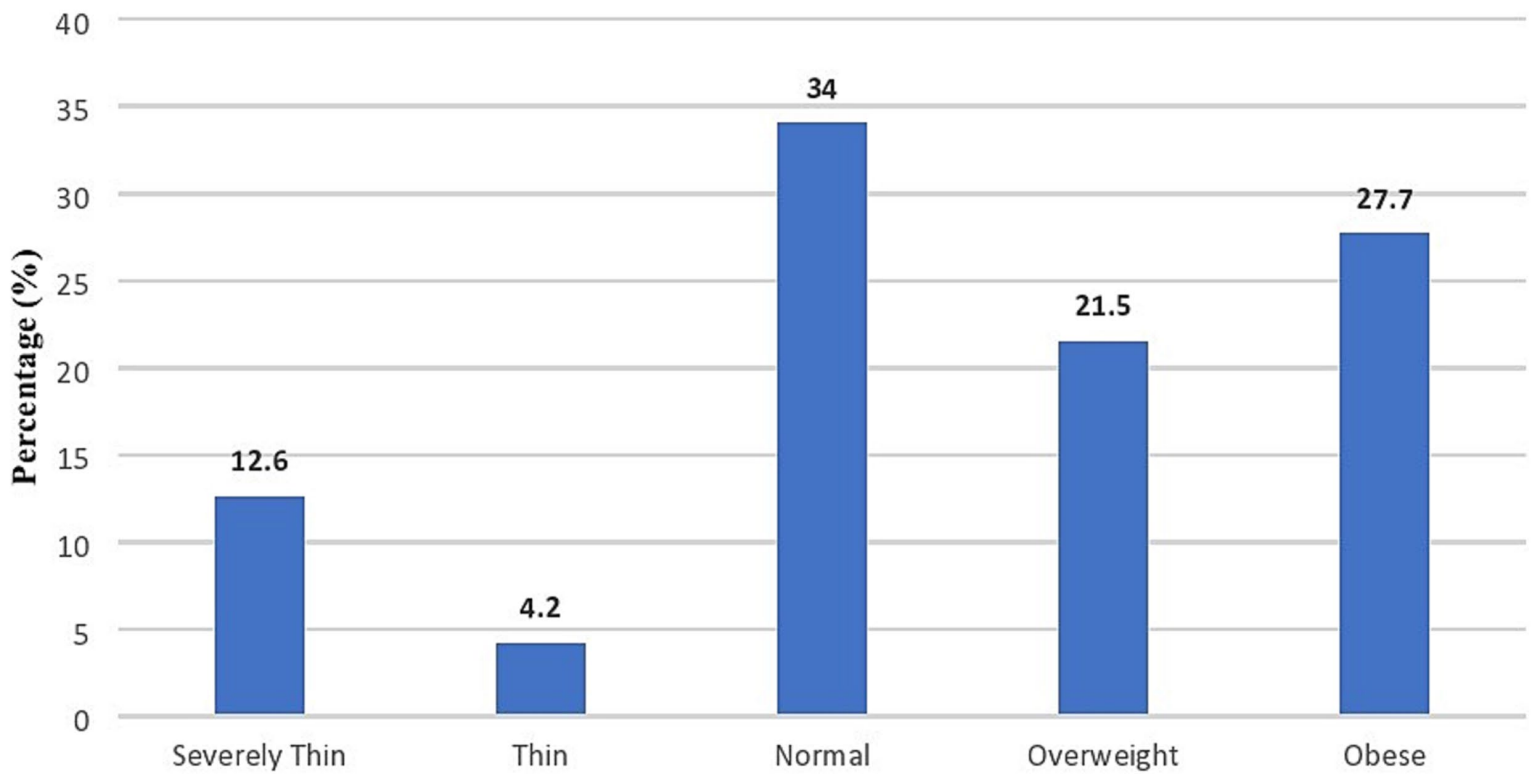
BMI of children.

In our extensive investigation into childhood obesity within the context of Pakistan, we meticulously scrutinized a spectrum of factors encompassing demographic characteristics, knowledge, attitudes, and practices (KAP) among our cohort of mothers. Our findings, elucidated in Table 1, provide nuanced insights into the landscape of childhood obesity in the region. Notably, a substantial proportion of our study participants (46.1%) fell within the age bracket of 31 to 40 years. Moreover, nearly half of the respondents (48.7%) had pursued educational levels beyond intermediate. Additionally, a predominant majority (86.9%) identified as housewives, while a majority hailed from urban locales. Furthermore, our cohort consisted of diverse financial backgrounds. The distribution of Body Mass Index (BMI) labels among the index children in the study cohort is depicted in Figure 1. The majority of children fell within the normal BMI range. A significant proportion of children were classified as obese (27.7%) or overweight (21.5%). Conversely, a quite smaller percentage of children were categorized as thin or severely thin.

**Table 1:** Comparison of knowledge, attitude, and practice with socio-demographic profile.

Characteristics	N (%)	Knowledge		Attitude		Practice	
		Percentage (%)	Correlation	Percentage (%)	Correlation	Percentage (%)	Correlation
**Age (years)**				*	**	*	
21–30	87(45.5)	63.1	−0.10	77.4	0.14	70.2	−0.04
31–40	88 (46.1)	58.9		79.0		70.8	
>40	16 (8.4)	55.2		81.3		64.9	
**The educational level of mother**		**	**		*	**	**
Illiterate	34 (17.8)	51.2	0.36	76.3	0.15	74.6	−0.26
Intermediate or below	64 (33.5)	54.6		77.9		71.0	
Above intermediate	93 (48.7)	68.0		79.6		67.8	
**Occupation of mother** ^**¥**^							
Housewife	166 (86.9)	59.7	0.10	78.1	0.14	70.2	−0.02
Working Women	25 (13.1)	66.0		81.2		69.4	
**Mother background** ^**¥**^		**	*	**	*	**	**
Urban	139 (72.8)	62.8	−0.19	79.5	−0.20	68.1	0.37
Rural	52 (27.2)	54.3		75.8		75.4	
**Monthly income of household (Rs)**		*	**	**	**	*	**
< 20,000	35 (18.3)	53.3	0.21	73.0	0.26	75.4	−0.30
21,000 -- 40,000	46 (24.1)	57.1		79.8		71.3	
41,000 -- 60,000	35 (18.3)	63.8		79.9		67.8	
61,000--80,000	41 (21.5)	66.7		78.8		69.0	
81,000 --100,000	22 (11.5)	62.9		80.3		67.4	
101,000 --120,000	5 (2.6)	66.7		83.2		64.8	
>121,000	7 (3.7)	54.8		79.1		65.0	
**Number of children**		**	**	*	*		
1–2	60 (31.4)	64.2	−0.17	79.4	−0.13	69.0	0.09
3–4	101 (52.9)	61.4		78.8		70.0	
>4	30 (15.7)	50.3		75.7		72.4	
**Number of people living in the household**		*	**				
2–4	52 (27.2)	66.0	−0.24	79.3	−0.05	69.8	−0.07
5–7	120 (62.8)	60.1		78.1		70.2	
>7	19 (9.9)	47.8		78.3		70.2	
**Child age (years)** ^**¥**^							
5–9	139 (72.8)	60.4	−0.02	78.2	−0.08	70.2	0.00
10–15	52 (27.2)	60.7		79.2		69.6	
**Child gender** ^**¥**^							
Male	105 (55)	59.8	0.06	79.0	−0.05	69.8	0.05
Female	86 (45)	61.4		77.8		70.4	
**BMI label**			**	**		**	**
Normal	65 (34)	59.7	0.17	79.8	−0.10	72.2	−0.21
Overweight	41 (21.5)	59.6		75.6		69.3	
Obese	53 (27.7)	66.0		78.0		66.5	
Thin	8 (4.2)	55.2		79.8		77.6	
Severely thin	24 (12.6)	53.8		80.5		71.0	

*p < 0.05 was considered statistically significant.

**p < 0.01 was considered statistically significant.

^¥Mann Whitney U test.^

**Figure 2:**
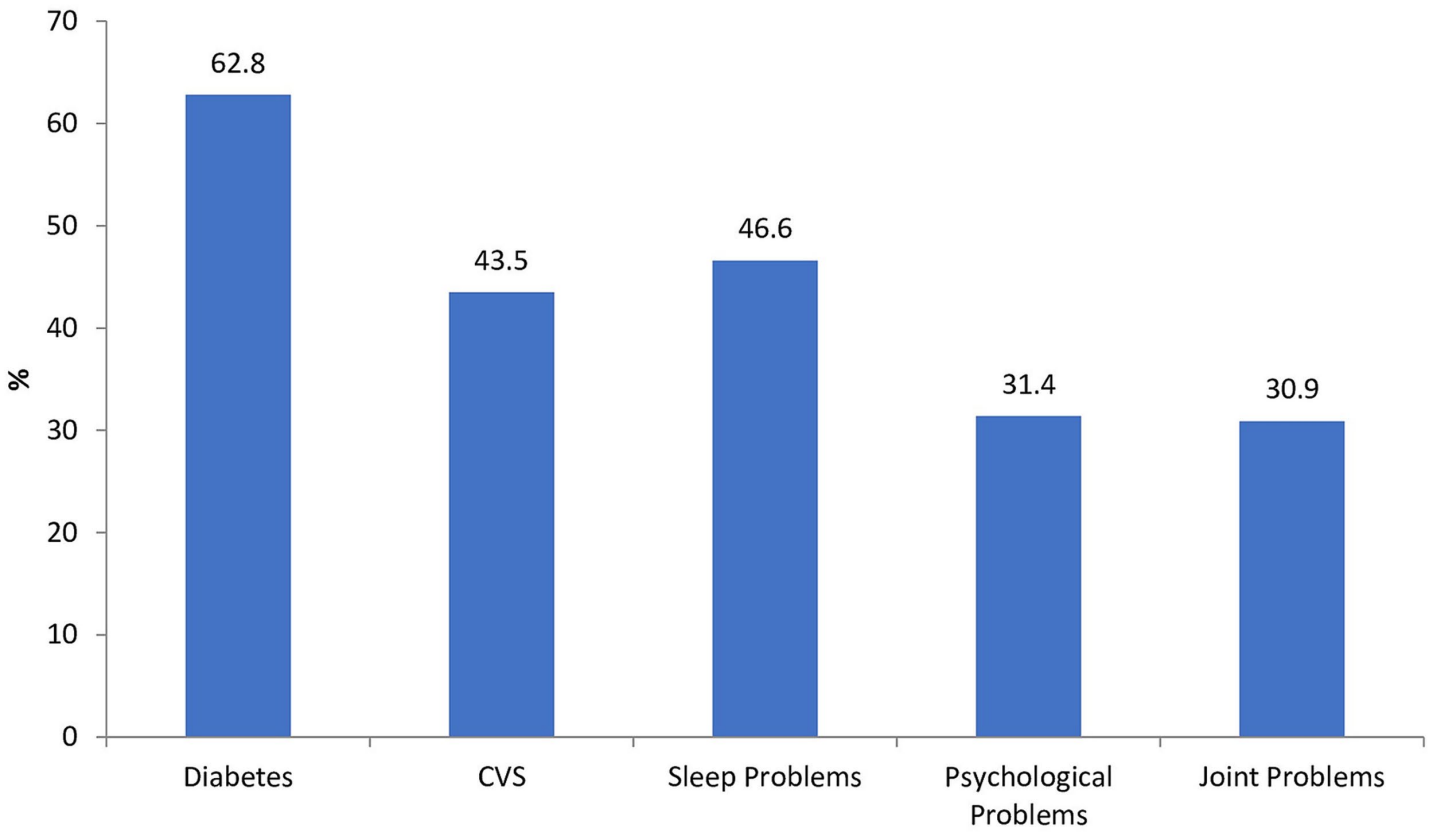
Do you think being overweight during childhood leads to problems later in life?

**Figure 3:**
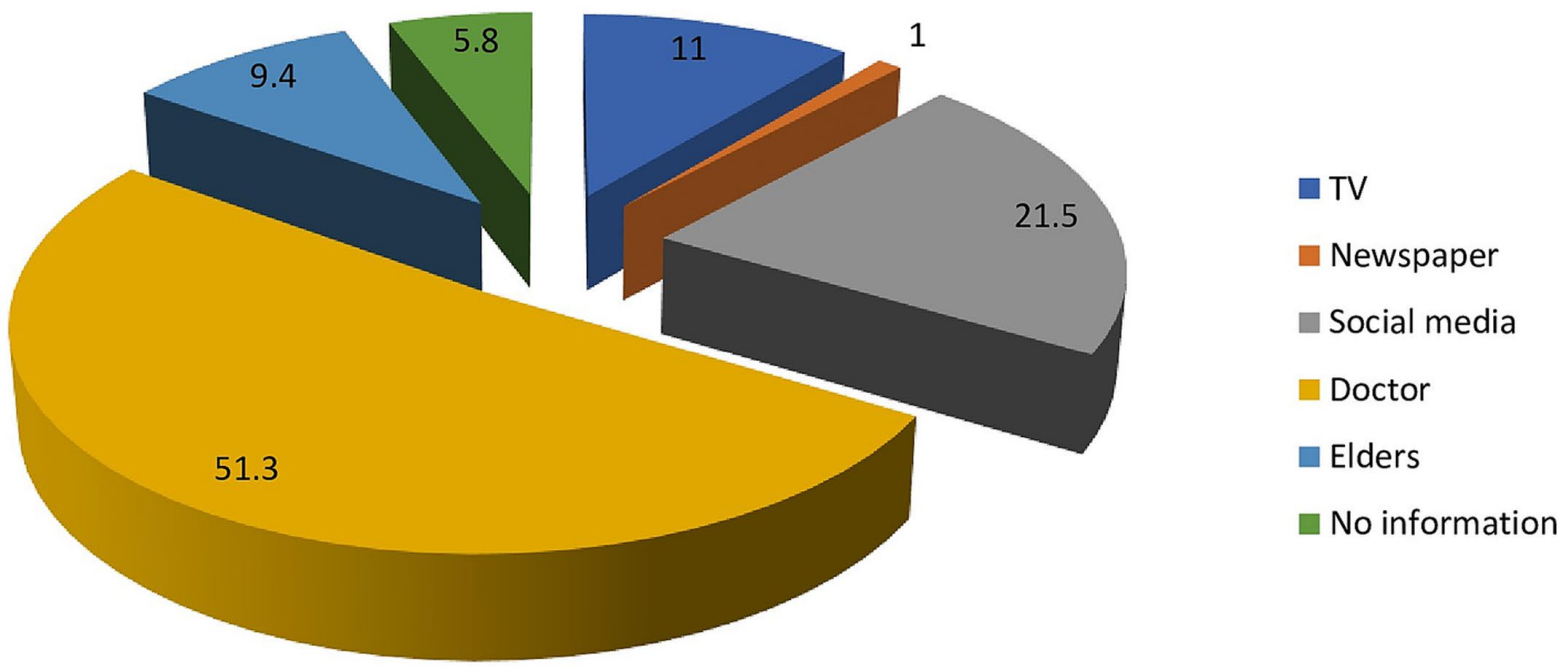
Where do you get your information about childhood obesity?

We measured mothers’ knowledge of childhood obesity and based on the scores; mothers demonstrated a moderate level of knowledge (60.5%) about childhood obesity. In terms of health impact related to childhood obesity, 75.4% of participants believed that being overweight during childhood could lead to health problems later in life. Furthermore, as depicted in Figure 2, a substantial proportion of mothers, constituting 62.8%, associated childhood obesity with the development of diabetes. Furthermore, 43.5% identified cardiovascular diseases (CVD) as a potential consequence of childhood obesity, moreover, they also recognized its association with other health outcomes. Interestingly, 37.2% believed that childhood obesity naturally disappears as the child grows up. When presented with a series of images, 62.8% selected the right image as representative of a healthy child. The pie chart in Figure 3 highlights the primary channels through which mothers acquire information about childhood obesity in Pakistan. Notably, the two major sources identified are doctors and social media. Our findings revealed a positive association between mothers’ knowledge of childhood obesity and several factors. Mothers with higher educational levels demonstrated a moderate positive correlation with knowledge of childhood obesity. Furthermore, a weak positive correlation was observed with both the household’s monthly income and the child’s BMI. Lastly, a weak negative correlation emerged between knowledge of childhood obesity and three other factors: the mother’s background, the number of children, and the total number of people the mother cares for.

**Figure 4:**
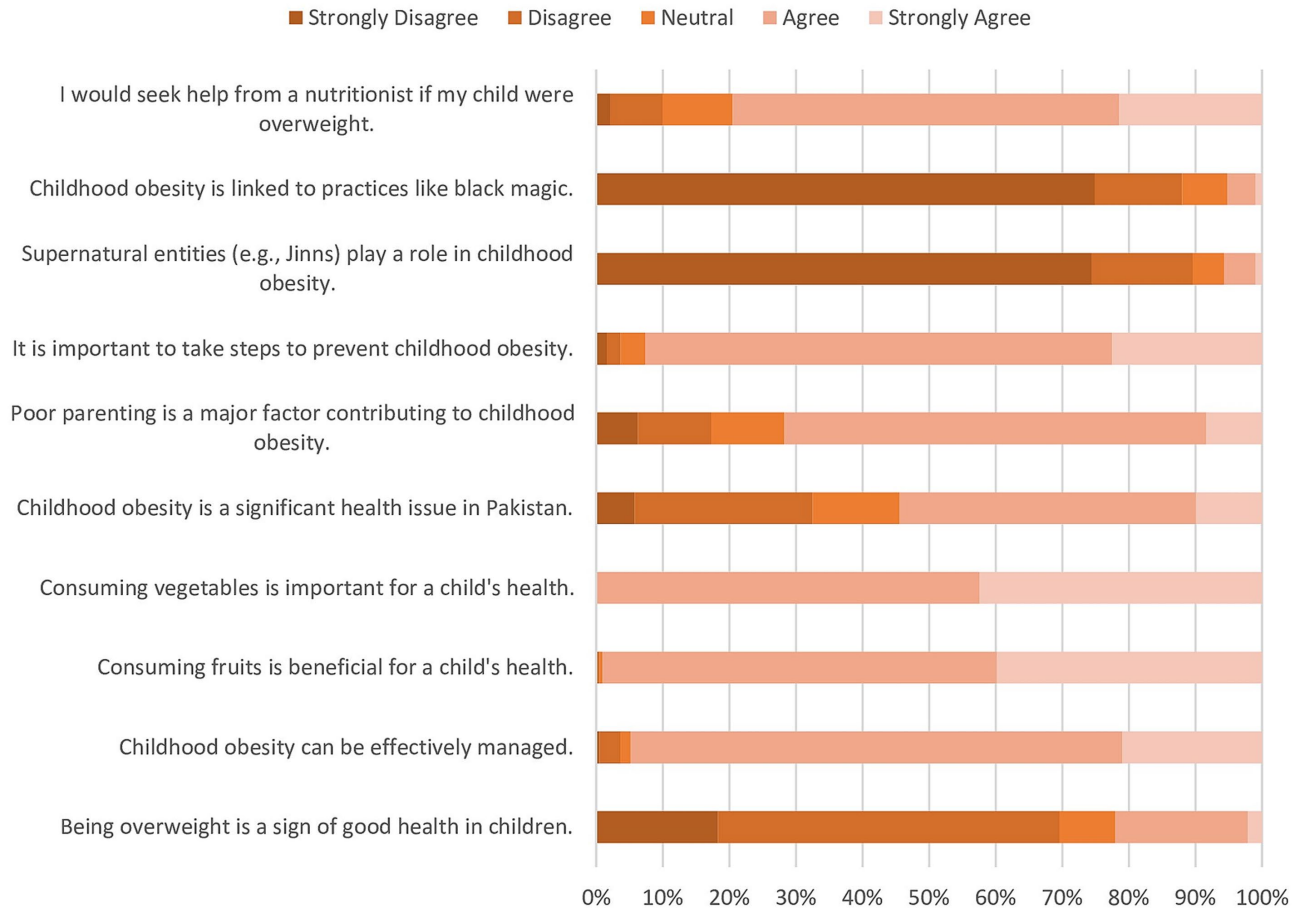
Descriptive on attitude related to childhood obesity?

Attitudes toward childhood obesity among mothers in Pakistan were assessed across various dimensions. Mothers demonstrated a notably positive outlook with a high level of agreement with the importance of healthy weight, reflected in their average score of 78.5% on the attitude assessment. The findings, presented in Figure 4, delineate the distribution of responses across different levels of agreement. A substantial proportion of respondents expressed concern about the health implications of childhood obesity. Notably, a majority (51.3%) disagreed with the notion that an overweight child is healthier. Similarly, most respondents (73.8%) agreed that obesity can be controlled. Regarding dietary perceptions, a notable proportion (39.8% & 42.4%) expressed neutrality regarding the health benefits of fruits and vegetables, respectively. Opinions on the prevalence and etiology of childhood obesity varied among respondents. While a considerable number acknowledged childhood obesity as a problem in Pakistan (44.5% agreed), fewer attributed it to poor parenting (8.4% agreed). Intriguingly, a considerable number of respondents distanced themselves from supernatural explanations for childhood obesity. A substantial majority did not associate childhood obesity with supernatural entities such as Jinns (74.3%) and black magic (74.9%). A significant moderate positive correlation emerged between mothers’ attitudes and the household’s monthly income. Mothers’ age and education level also showed a significantly weak positive correlation with attitudes, and interestingly, a weak negative correlation was observed between attitudes and both mother’s background and the number of children in the household.

Mothers also displayed encouraging practices related to promoting healthy weight in their children, with an average score of 70.1% on the practices assessment. Table 2 provides a descriptive overview of practices related to childhood obesity among mothers in Pakistan. The majority of children were reported to spend considerable time using electronic devices daily, with 67% spending 1–3 h daily. Regarding sleep duration, a significant proportion of children received 7–11 h of sleep each night (93.2%). In terms of physical activity, a noteworthy finding is that the largest proportion of children engaged in physical activity or exercise for 1–2 h per day (51.3%). The consumption of margarine or butter was reported by the majority of mothers (51.3%). In terms of dietary habits, a substantial proportion of children consumed processed foods, sugary drinks, and fast food all sometimes but not every day (64.9, 62.3, and 56% respectively). In terms of correlation, Both the increase in BMI of the index child and the Education of the mother showed a significantly weak negative correlation with practices. Having a lower education status resulted in better practices, thus in turn healthier children in terms of BMI. Moreover, mothers from rural backgrounds had better practices compared to their urban counterparts.

**Table 2:** Distribution of responses across practices section.

**Questions**		***N* (%)**
How much time does your child spend using electronic devices each day, such as phones, tablets, and computers?	< 1 h	36 (18.8)
	1--2 h	71 (37.2)
	2--3 h	57 (29.8)
	3--4 h	12 (6.3)
	> 4 h	15 (7.9)
How many hours of sleep does your child get each night?	5--7 h	13 (6.8)
	7--9 h	95 (49.7)
	9--11 h	83 (43.5)
How much time does your child spend engaged in physical activity or exercise each day?	< 30 min	11 (5.8)
	30--60 min	30 (15.7)
	1--2 h	98 (51.3)
	2--3 h	42 (22)
	> 3 h	10 (5.2)
Does your child consume margarine or butter regularly?	Yes	98 (51.3)
	No	93 (48.7)
Does your child regularly consume fruits?	No	56 (29.3)
	Yes	135 (70.7)
Does your child regularly consume vegetables?	No	90 (47.1)
	Yes	101 (52.9)
How often does your child consume processed or packaged food?	Everyday	38 (19.9)
	Not every day but sometimes	124 (64.9)
	Not at all	29 (15.2)
How often do you give your child sugary drinks?	Everyday	56 (29.3)
	Not every day but sometimes	119 (62.3)
	Not at all	16 (8.4)
How often does your child consume fast food or junk food items in a typical week?	Everyday	51 (26.7)
	Not every day but sometimes	107 (56)
	Not at all	33 (17.3)

Lastly, among the surveyed mothers, a significant majority (90.1%, *n* = 172) acknowledged the existence of stigmatization toward underweight children within the Pakistani societal context. Conversely, a minority (9.9%, *n* = 19) reported otherwise. Furthermore, an overwhelming majority of respondents (96.9%, *n* = 185) indicated that they had either personally witnessed or experienced instances of stigmatization directed toward obese children. In contrast, only a small fraction (3.1%, *n* = 6) reported no such encounters. A notable proportion of mothers (79.6%, *n* = 152) admitted to feeling pressured by relatives to perceive their child as underweight. Conversely, a minority (20.4%, *n* = 39) reported no such pressure.

## 4 Discussion

Childhood obesity poses a significant public health challenge globally, and Pakistan is no exception. Our study sought to delve into the intricate landscape of childhood obesity in Pakistan by elucidating its prevalence and the associated knowledge, attitudes, and practices among mothers, who play a pivotal role in shaping the dietary and physical activity behaviors of their children. Our findings shed light on the intricate interplay of sociodemographic factors, maternal perceptions, and health-related behaviors contributing to the obesity epidemic in Pakistan.

Pakistan, undergoing a transitional phase, confronts the dual challenge of simultaneous overnutrition and undernutrition. Our study revealed a notable prevalence of childhood obesity, with 27.7% of Pakistani children classified as obese in addition to 21.5% of overweight children. It’s important to note that rates of childhood obesity vary across regions and populations, however, these findings align with the rising trend of childhood obesity observed globally (14), but it surpasses figures reported in previous studies conducted between 2008 to 2022 within Pakistan ranging from 6 to 12% (4, 15–17). This significant disparity highlights the urgency for further investigation into the specific factors contributing to the epidemic of childhood obesity in Pakistan (7).

Notably, our findings indicate that mothers demonstrate a moderate level of knowledge, with 60.5% displaying an understanding of childhood obesity. Of particular significance is the association between childhood obesity and the development of chronic diseases, such as diabetes and cardiovascular diseases (CVD), as perceived by Pakistani mothers. While this level of knowledge indicates a reasonable awareness among mothers, there are notable implications for public health interventions and strategies aimed at addressing childhood obesity in Pakistan. In contrast, studies from high-income countries (HICs) often report higher levels of maternal knowledge regarding childhood obesity (18). This disparity may be attributed to differences in access to healthcare, educational resources, and socioeconomic factors. Nonetheless, the findings underscore the universal importance of maternal education as a key determinant of childhood obesity prevention and management, regardless of geographical location.

One key finding was that only 62.8% of mothers correctly identified an image depicting a healthy child. This highlights a potential knowledge gap regarding healthy weight ranges for children. Misconceptions about ideal body size can lead to underestimating childhood obesity and neglecting its health risks (19). This aligns with research, where one-third of parents held inaccurate perceptions of their child’s weight, with the majority (93%) underestimating it (20). However, it is important to note that studies in Pakistan have documented high rates of childhood stunting (21), which can coexist with overweight and obesity. Mothers’ focus might be on ensuring adequate child growth, potentially hindering recognition of childhood overweight and obesity (22). Further research is needed to explore this possibility.

One key finding of our study is the notably positive outlook demonstrated by mothers toward the importance of maintaining a healthy weight. This is reflected in their average score of 78.5% on the attitude assessment, indicating a strong agreement with the significance of healthy weight for children. This positive outlook is further supported by the fact that a majority disagreed with the misconception that overweight children are healthier. Thus, this positive attitude is encouraging and suggests a readiness among mothers to prioritize the health and well-being of their children. Additionally, it is important to investigate whether this positive attitude translates into appropriate practices to prevent childhood obesity.

In Pakistan, cultural beliefs deeply intertwine with perceptions of health and illness. Many Pakistanis attribute health problems to supernatural causes such as the influence of jinn or black magic. These beliefs stem from religious teachings and cultural traditions (23). The association of health problems with black magic or Jinns can profoundly impact health practices, including treatment-seeking behavior. Individuals may prioritize seeking spiritual or religious remedies over medical interventions, potentially delaying or hindering appropriate healthcare access. Intriguingly, our study revealed that a significant portion of respondents in Pakistan tend to reject supernatural explanations for childhood obesity. Despite prevalent beliefs in supernatural causes of health issues like obesity in Pakistan, a noteworthy segment of the population appears to challenge these traditional notions. This suggests a potential shift in cultural attitudes toward more evidence-based explanations for health conditions.

Parental encouragement and caring about healthy eating significantly influence children’s diet quality and reduce the likelihood of being overweight. This aligns with the observed high engagement of mothers in health-promoting practices, as parental involvement is critical in shaping children’s health behaviors (24). Our findings indicate that mothers displayed an average score of 70.1% on practices related to childhood obesity, with lower education status and rural backgrounds surprisingly linked to better practices. These results might be influenced by the socioeconomic context of the sample, which predominantly consists of low- to middle-income families. The finding that lower education status is associated with better practices is counterintuitive but can be understood through the lens of specific community dynamics. In some low- to middle-income and rural settings, there may be a stronger reliance on traditional practices and community support systems that promote healthier lifestyles. For instance, rural areas often have more opportunities for physical activity through outdoor play and community-based sports. Moreover, lower education status might correlate with more time spent at home, leading to greater parental involvement in children’s daily activities, including meal preparation and active play. Similarly, the result that mothers from rural backgrounds exhibited better practices may reflect the benefits of a lifestyle that is less urbanized and potentially more active. Rural communities often have closer-knit social structures that can support healthy behaviors through shared community norms and activities. Additionally, rural living can naturally involve more physical labor and outdoor activities, contributing to a healthier lifestyle for children. However, it is important to note that other studies, such as a recent investigation into the Mediterranean Diet, found a positive association between parental educational level and healthier dietary adherence in children (25). This highlights that while our findings suggest lower education status may be linked to better practices in certain rural contexts, the broader literature emphasizes the role of education in promoting healthier food choices. It is possible that the unique socio-cultural dynamics in rural Pakistan mediate the influence of education on health behaviors differently than in more urbanized or Western settings.

The overwhelming majority of respondents (96.9%, *n* = 185) indicating that they had either personally witnessed or experienced instances of stigmatization directed toward obese children is a significant and concerning finding. This high prevalence of reported stigmatization underscores the pervasive nature of weight bias and its impact on the well-being of children. Stigmatization of obese children can have far-reaching consequences on their physical health, psychological well-being, and social development. Weight-based stigma can manifest in various forms, including teasing, bullying, and social exclusion, which can lead to detrimental outcomes such as low self-esteem, depression, and anxiety (26). The psychological distress caused by such experiences can also contribute to unhealthy behaviors, including disordered eating and avoidance of physical activity, further exacerbating the problem of obesity. This vicious cycle highlights the importance of addressing weight stigma as a public health priority (27).

Our study on exploring the knowledge, attitudes, and practices of mothers provides critical insights but also highlights areas necessitating further research and certain limitations. Future studies should aim for larger, more diverse sample sizes to enhance generalizability across different socio-economic and cultural contexts. Longitudinal studies would be beneficial to understand how maternal knowledge and practices evolve over time and their long-term impact on child health outcomes. Additionally, incorporating mixed-method approaches could enrich the data, providing both quantitative breadth and qualitative depth. However, our study faced limitations such as potential self-reporting biases and the cross-sectional design, which restricts the ability to infer causality. Furthermore, the reliance on self-administered questionnaires may have limited the depth of understanding of mothers’ practices and attitudes. Fathers, as important figures in the family structure, can significantly influence children’s health behaviors alongside mothers. Studies show that fathers often recognize their role in shaping their children’s diet and activity levels, which are key factors in obesity risk (28). However, there can sometimes be a disconnect between their positive beliefs about healthy eating and their practices, such as frequent eating out, which promotes a more sedentary lifestyle (29). Therefore, the involvement of fathers or other parental figures should be considered when assessing the family’s overall impact on childhood obesity. Addressing these limitations in future research will be crucial for developing more effective maternal and child health interventions.

## 5 Conclusion

In conclusion, our study highlights the significant challenge of childhood obesity in Pakistan, with 27.7% of children classified as obese and 21.5% as overweight. This prevalence, exceeding previous national reports, calls for urgent action to address the contributing sociodemographic and cultural factors. While 60.5% of mothers demonstrate a moderate understanding of obesity and its health risks, gaps remain in recognizing healthy weight ranges. Positively, mothers show strong attitudes toward maintaining healthy weights and engaging in health-promoting practices, though lower education and rural backgrounds unexpectedly correlate with better practices. The high incidence of stigmatization toward obese children, reported by 96.9% of respondents, underscores the need to address weight bias. Our findings emphasize the importance of culturally sensitive, evidence-based public health initiatives to combat childhood obesity and promote healthier futures for Pakistani children. To improve mothers’ understanding of their child’s weight in Pakistan, community-based educational workshops should focus on body mass index (BMI) and healthy growth patterns, supported by regular pediatric visits for personalized assessments. Culturally tailored public health campaigns and structured behavioral change programs can enhance mothers’ attitudes toward affordable nutrition and physical activity. Involving fathers and extended family members in these initiatives fosters a supportive environment that encourages healthier choices for children.

## Data Availability

The raw data supporting the conclusions of this article will be made available by the authors, without undue reservation.
