# Supplementary material for: Understanding childhood obesity in Pakistan: exploring the knowledge, attitudes, practices of mothers, and influential factors. A cross-sectional study
Source: Front Public Health. 2024 Nov 6;12:1475455. doi: 10.3389/fpubh.2024.1475455 (PMC11576336; doi:10.3389/fpubh.2024.1475455)
Supplement: Supplementary file 1 [file Table_1.DOCX]

**Questionnaire**

**Section 1: Demographic info:**

1. Age of the mother

|  |
| --- |

2. Education level of the mother

| Illiterate | Intermediate or below | Above intermediate |
| --- | --- | --- |

3. Occupation of the mother

|  |
| --- |

4. Mother’s Background

| Urban | Rural |
| --- | --- |

5. Monthly income of the household

| < =20,000 | 21,000 – 30,000 | 30,000 – 40,000 | 41,000-50,000 | >50,000 |
| --- | --- | --- | --- | --- |

6. Number of children in the household

|  |
| --- |

7. Number of family members living in the household

|  |
| --- |

8. Child age (between 5 to 15)

|  |
| --- |

9. Gender of the Child

| Male | Female | Other |
| --- | --- | --- |

**Section 2: Maternal Knowledge of Childhood Obesity:**

1. Do you think being overweight during childhood can lead to health problems later in life?

| Yes | No | Maybe |
| --- | --- | --- |

2. What are the health consequences of childhood obesity?

| Diabetes | CVS Problems | Sleep Problems | Joint Problems | Psychological issues |
| --- | --- | --- | --- | --- |

3. According to you, is obesity something that runs in the family?

| Yes | No | Maybe |
| --- | --- | --- |

4. Do you think obesity goes away once the child grows up?

| Yes | No | Maybe |
| --- | --- | --- |

6. What do you think about your child's weight?

| Underweight | Normal | Overweight |
| --- | --- | --- |

7. According to you, which child from the following pictures is healthy?


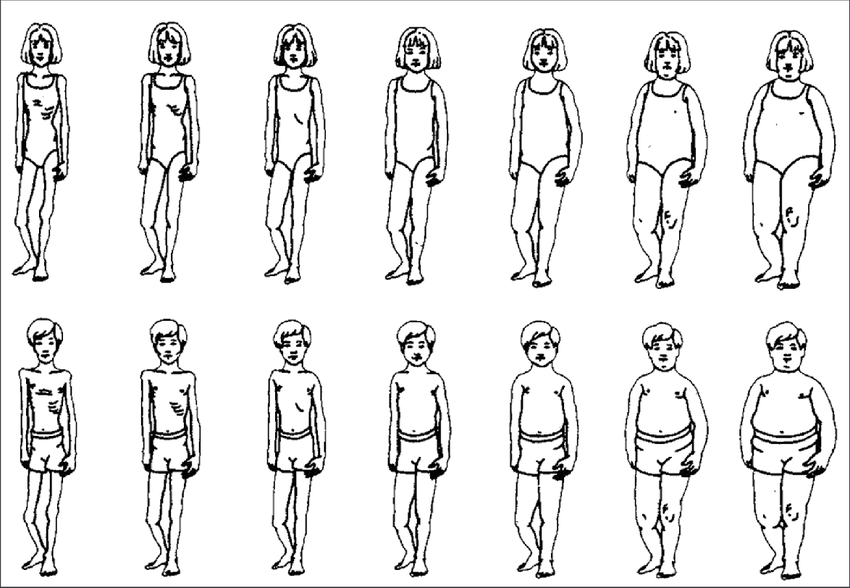


**Section 3: Attitudes towards childhood obesity:**

|  | ***Strongly Disagree*** | ***Disagree*** | ***Neutral*** | ***Agree*** | ***Strongly Agree*** |  |
| --- | --- | --- | --- | --- | --- | --- |
|  |  |  |  |  |  |  |
| Being overweight is a sign of good health in children. |  |  |  |  |  |  |
| Childhood obesity can be effectively managed. |  |  |  |  |  |  |
| Consuming fruits is beneficial for a child's health. |  |  |  |  |  |  |
| Consuming vegetables is important for a child's health. |  |  |  |  |  |  |
| Childhood obesity is a significant health issue in Pakistan. |  |  |  |  |  |  |
| Poor parenting is a major factor contributing to childhood obesity. |  |  |  |  |  |  |
| It is important to take steps to prevent childhood obesity. |  |  |  |  |  |  |
| Supernatural entities (e.g., Jinns) play a role in childhood obesity. |  |  |  |  |  |  |
| Childhood obesity is linked to practices like black magic. |  |  |  |  |  |  |
| I would seek help from a nutritionist if my child were overweight. |  |  |  |  |  |  |

**Section 4: Practices related to childhood Obesity:**

1. How much time does your child spend using electronic devices each day, such as phones, tablets, and computers?

| <1 hour | 1-2 hour | 2-3 hour | 3-4 hour | >4 hour |
| --- | --- | --- | --- | --- |

1. On average, how much time does your child spend studying each week?

| <1 hour | 1-3 hour | 3-5 hour | 5-7 hour | >7 hour |
| --- | --- | --- | --- | --- |

1. On average, how many hours of sleep does your child get each night?

| <5 hour | 5-7 hour | 7-9 hour | 9-11 hour | >11 hour |
| --- | --- | --- | --- | --- |

1. On average, how much time does your child spend engaged in physical activity or exercise each day?

| <30 minutes | 30-60 minutes | 1-2 hour | 2-3 hour | >3 hour |
| --- | --- | --- | --- | --- |

1. What is the usual number of meals that your child eats each day?

| 1 | 2 | 3 | 4 | 5 |
| --- | --- | --- | --- | --- |

1. Does your child consume margarine or butter regularly?

| Yes | No |
| --- | --- |

1. On average, how many chapattis or other bread items does your child consume in a typical day?

| 1 | 2 | 3 | 4 | 5 | 6 |
| --- | --- | --- | --- | --- | --- |

1. Does your child regularly consume fruits and vegetables

| yes | No |
| --- | --- |

1. How often does your child consume processed or packaged food?

| Everyday | Not Everyday but sometimes | Not at all |
| --- | --- | --- |

1. How often do you give your child sugary drinks or juices?

| Everyday | Not everyday but sometimes | Not at all |
| --- | --- | --- |

1. How often does your child consume fast food or junk food items in a typical week?

| Everyday | Not everyday but sometimes | Not at all |
| --- | --- | --- |

1. What steps do you take to promote healthy habits and prevent childhood obesity in your child?
2. Do you encourage your child to participate in physical activities or sports? If so, what activities?

**Section 5: Perception of Obese Children and Stigmatization:**

1. Do you believe that underweight children are stigmatized in Pakistan?

| Yes | No |
| --- | --- |

2. Have you ever witnessed or experienced the stigmatization of obese children?

| Yes | No |
| --- | --- |

3. What do you think are the consequences of the stigmatization of obese children?

4. Have you ever felt pressured by relatives into thinking that your child is underweight?

| Yes | No |
| --- | --- |

**Section 6: Sources of Information:**

1. Where do you get information about childhood obesity?

| TV | Newspaper | Social Media | Doctor |
| --- | --- | --- | --- |

2. Do you think the information available about childhood obesity is sufficient?

| Yes | No |
| --- | --- |
